# A Theoretical Model to Predict Both Horizontal Displacement and Vertical Displacement for Electromagnetic Induction-Based Deep Displacement Sensors

**DOI:** 10.3390/s120100233

**Published:** 2011-12-28

**Authors:** Nanying Shentu, Hongjian Zhang, Qing Li, Hongliang Zhou, Renyuan Tong, Xiong Li

**Affiliations:** 1 State Key Laboratory of Industry Control Technology, Zhejiang University, Hangzhou, Zhejiang 310027, China; E-Mails: stnying_2@163.com (N.S.); hjzhang@iipc.zju.edu.cn (H.J.Z.); zjuzhl@zju.edu.cn (H.L.Z.); 2 College of Information Engineering, China Jiliang University, Hangzhou, Zhejiang 310018, China; 3 College of Mechatronics Engineering, China Jiliang University, Hangzhou, Zhejiang 310018, China; E-Mails: tongrenyuan@163.com (R.T.); lixiong@cjlu.edu.cn (X.L.)

**Keywords:** electromagnetic induction-based deep displacement sensor, theoretical modeling, deep horizontal displacement, deep vertical displacement, mutual inductance

## Abstract

Deep displacement observation is one basic means of landslide dynamic study and early warning monitoring and a key part of engineering geological investigation. In our previous work, we proposed a novel electromagnetic induction-based deep displacement sensor (I-type) to predict deep horizontal displacement and a theoretical model called equation-based equivalent loop approach (EELA) to describe its sensing characters. However in many landslide and related geological engineering cases, both horizontal displacement and vertical displacement vary apparently and dynamically so both may require monitoring. In this study, a II-type deep displacement sensor is designed by revising our I-type sensor to simultaneously monitor the deep horizontal displacement and vertical displacement variations at different depths within a sliding mass. Meanwhile, a new theoretical modeling called the numerical integration-based equivalent loop approach (**NIELA**) has been proposed to quantitatively depict II-type sensors’ mutual inductance properties with respect to predicted horizontal displacements and vertical displacements. After detailed examinations and comparative studies between measured mutual inductance voltage, **NIELA**-based mutual inductance and EELA-based mutual inductance, **NIELA** has verified to be an effective and quite accurate analytic model for characterization of II-type sensors. The **NIELA** model is widely applicable for II-type sensors’ monitoring on all kinds of landslides and other related geohazards with satisfactory estimation accuracy and calculation efficiency.

## 1. Introduction

Landslides occur in many areas in the World, causing not only heavy property losses but also serious loss of human lives. Landslide deformation is an integrated reflection of geological structure and internal and external influencing factors of a landslide mass. Therefore, landslide deformation monitoring is a basis for the analysis of the geological structure and dynamic deformation characteristics of the investigated landslide mass, a support for informatization design of sliding remediation projects, and a promise of feasible technology to predict and provide advance warning against geo-hazards [1–5]. Landslide deformation monitoring is based on displacement measurement information and mainly includes surface displacement monitoring and deep displacement monitoring [6–8]. Compared to the former, landslide deep displacement monitoring is more complicated while more significant, because through it, the landslide deformation mode could be deduced, the sliding plane location and depth decided, and the dynamic ranges and trends of sliding deformation judged, thereby providing a scientific basis and reliable information for the analysis of a landslide’s stability conditions, deformation mechanics and the related design of treatment engineering [9,10].

On the other hand, both surface displacement monitoring and deep (or subsurface) displacement monitoring have two aspects: horizontal displacement monitoring and vertical displacement monitoring. The significance of measuring and monitoring these two aspects simultaneously has been fully demonstrated and verified by lots of theoretical research and engineering cases concerning landslides and other slope movements.

Surface displacement monitoring [11–13], whether in terms of surveying techniques or monitoring instrumentation has developed rapidly and reached a high level. The conventional instrumentation for surface displacement monitoring include settlement gauges, precision levelings, theodolites, surface extensometers for surface vertical displacement measurement, ultrasonic or laser distance meters, and deflectometers for horizontal displacement measurement, and total station, aerial photogrammetry for measurement in both directions. The modern techniques [14–16] for surface displacement monitoring include multi-antenna GPS receiver, DInSAR (Differential Interferometric Synthetic Aperture Radar), terrestrial laser scanning, *etc*.

Compared to the surface displacement monitoring, development of deep displacement monitoring [17,18], whether in terms of monitoring techniques, methods or instrumentations (including related sensors) is much slower thus has greatly limited its application scope and popularity, due to the extremely complicated and variable characteristics of deep rock and soil mass, such as temporal-paroxysmic, spatial-randomicity and invisibility, conditional-terribleness (e.g., corrosion, seepage of underground water, failures of shear and compression), geological-heterogeneity and complexity.

At present, methods/instruments for deep displacement monitoring can mainly grouped to three categories. The first one is borehole extensometers, which includes two subcategories: multi-point borehole extensometers [19,20] and wire/cable extensometers [21]. Although wire/cable extensometers are relatively simple and low-cost devices, displacements measured by them are global (*i.e*., the total changing distance from one point on landslide surface to another fixed point inside the borehole below the slip surface) and the device can neither detect the vertical components and horizontal components of the underground slope movement, nor identify the presence of several slip surfaces, nor obtain the relative displacements at different depths within the sliding mass. Multi-point borehole extensometers are conventional devices to monitor the change of vertical displacements at pre-selected depth that decided by pre-installed targets along the common axis of vertical borehole, so they are mainly used for settlement and heave monitoring of underground soil and rock. The main disadvantages of borehole extensometers include the fact that instrument installation is difficult and complex under deep borehole conditions, sliding surfaces are hard to determine, rods/probes may be wedged if large lateral displacement occurs, and data reading is laborious.

The second one is slope inclinometers [22–24]. Presently, they are widely applied to monitor deep horizontal displacement at a constant interval of depth within the slope mass and to locate the potential sliding surfaces. Although slope inclinometers work based on a relatively simple principle, they do not allow continuous recording of displacement, so it is hard to monitor landslides in real-time and automatically. Furthermore, they are vulnerable to fail or “shear off” due to jams, S-shape or over-large displacements, cut-off of inclinotubes and other reasons.

The third one is Time Domain Reflectometry (TDR) [25–27], which uses an coaxial cable as a sensor and works like radar to locate the depth of shear planes or deformation zones in a landslide. TDR is a relatively low cost and time-saving monitoring approach, but it can hardly decide the sliding magnitude and direction of deep displacement, nor monitor landslides with heavy sliding bands. Furthermore, TDR cannot be used where a shear zone does not occur but monitoring tilt is necessary.

To summarize, at present there exist few sensors or instruments that can simultaneously and efficiently monitor the horizontal displacement and vertical displacement of subsurface deformation at different depths within the soil and rock mass.

In our preceding study [28], a novel electromagnetic induction-based integrated deep displacement sensor (hereafter called the I-type deep displacement sensor) was presented. It can directly convert the varied deep horizontal displacement and tilt angle at any depth within the sliding mass to the corresponding variation of mutual inductance between any two adjacent solenoids (each solenoid functions as a sensor unit), so it can monitor the underground horizontal displacement more conveniently and accurately with a relatively simple and low-cost design.

Theoretical modeling is an essential and important work in sensor design, error analysis, and optimization processes, as it can greatly help to understand the behavior of the sensor so as to optimize sensor design and solve some concurrent problems.

In our previous work [28], in order to describe the complicated relationship among the underground sliding mass’ horizontal displacement and tilt direction, the I-type sensor’s geometric parameters and its related output of mutual inductance and mutual inductance voltage, we have initially proposed a theoretical model called *equation-based equivalent loop approach* (**EELA**). Through a series of comparative studies between the experimental results based on our I-type sensor prototype and **EELA**-based theoretical simulation results, we not only initially showed the sensor’s feasibility, but also validated that the **EELA** model is quite suitable for depicting the said sensor’s sensing properties thanks to its commonality, effectiveness, and adequate accuracy.

We note that in our last paper [28], to adapt to the I-type sensor, it was assumed that along with the deep sliding movement, any two adjacent sensor units (labeled as Solenoid I and II) were free to relatively tilt and move horizontally, but no obvious vertical displacement occurred between them. The same assumption has been adopted in the **EELA** modeling process for an I-type deep displacement sensor. However the supposition that vertical displacement does not change or changes negligibly places great limitations on the deep displacement monitoring process of landslides and other geological disasters. That’s to say, in many monitoring cases for landslides and other slope movements, both the horizontal displacement and vertical displacement vary apparently and dynamically, and may require simultaneous monitoring of both, which can then provide more comprehensive and objective monitoring guidelines for the deep displacement monitoring process.

Let’s explain this in more detail. From the definition of landslide [29–31], both a generalized and narrow sense of landslide exists. The general sensor of “landslide” refers to “the movement of a mass of rock, debris, or earth down a slope” and mainly includes five types of mass movements: fall, topple, spread, slide and flow. The narrow sense refers only to “slides”, which mainly include two types: translational slides and rotational slides. Translational slides are mainly planarly displaced along the sliding surface, so it is reasonable to assume that no obvious vertical displacement occurs inside the sliding mass and to mainly monitor the horizontal displacement variations during the deep movement process. However, for a rotational landslide, especially during the compression creep stage, both vertical displacement and horizontal displacement change obviously and dynamically, so both may require monitoring. Moreover, for other landslide types and some related geohazards, especially for those caused by excessive underground coal mining, excessive groundwater extraction and slope foot cutting, a large amount of theoretical studies and engineering practices show that a simultaneous monitoring of deep horizontal displacement and vertical displacement is often required.

Therefore in this paper, some effective revisions have been made to the I-type deep displacement sensor both in the structure design and the correlated driver software, to make it meet the need for simultaneously monitoring the horizontal component and vertical component of landslide deep displacement. The revised version is then called a II-type deep displacement sensor.

In order to depict the mutual inductance properties of the proposed II-type sensor efficiently and accurately, a theoretical modeling called *numerical integration-based equivalent loop approach* (**NIELA**) is presented. Combining the numerical integration technique with the equivalent loop approach, this model can qualitatively and accurately evaluate the complicated relationships between the mutual inductance, the geometrical parameters of any two adjacent sensor units, and their relative position in terms of relative horizontal displacement, vertical displacement and axial angle, thereby both the variations of deep horizontal displacement and deep vertical displacement together with tilt directions at various depths within the monitored slope mass can be simultaneously monitored and quantitatively determined by a II-type deep displacement sensor. Modeling verification through experimental tests and comparative studies have confirmed the proposed **NIELA** model’s theoretical reliability and estimation accuracy in depicting the proposed sensor’s sensing properties.

## 2. Architecture and Principles of II-Type Deep Displacement Sensor

As previously described [28], an I-type deep displacement sensor is mainly constituted of a number of deep displacement measuring integrated sensor units in series (hereinafter referred to as sensor unit). Each sensor unit has identical structure, including an air-core solenoid as main component and embedded along its inner wall an integrated sensing circuitry PCB with such functions as sinusoidal voltage generation (*U_i_*), mutual inductance voltage measuring (*U_o_*), tilt angle measurement (*θ*_0_), RS485 bus communication with the deep displacement measuring central processing unit, *etc*.

Each sensor unit is vertically spaced a certain distance and encapsulated in a heat-shrinkable plastic soft tube, so forming a deep displacement measuring chain. By measuring the relative horizontal displacement and tilt angle variations between any two adjacent sensor units one by one, the cumulative deep horizontal displacement and sliding direction, from surface to different depths within the monitored sliding mass can be measured. Any two adjacent sensor units (Solenoid I and II) constitute a relatively deep displacement sensor that can be used for measuring the relative horizontal displacement and sliding angle at some given depth within the sliding mass.

Before an I-type sensor takes effect, these sensor units should be vertically buried into a borehole and backfilled tightly with cement grout so it can deform synchronously with the surrounding soil mass. It is worth noting that to fit the sensing properties of the I-type sensor [28], it is assumed that along with the deep sliding movement, any two adjacent sensor units are free to relatively tilt and move horizontally, but no obvious vertical displacement occurs between them. From the laws of electromagnetic mutual induction, when a sine voltage *U_i_* with fixed frequency and amplitude applied to solenoid I, a corresponding mutual induced voltage *U_o_* and mutual induction *M* will be generated across solenoid II (*M* has proved to be directly proportional to *U_o_*). Under the above assumptions, *U_o_* and *M* have a definitive functional relationship with the relative horizontal displacement *X* and axial angle *θ*_0_ between solenoid I and II, and their geometrical parameters in terms of diameter *D*, length *A* and winding coil turns *W*. So, the functional relation can be generally expressed as:

(1) $$U_{o}=f\;\left(X,\;\theta_{0},\;Z_{0},\;D,\;A,\;W\right)$$

where *Z*_0_ is the initial axial distance between Solenoid I and II, *θ*_0_ is their relative axial angle (equaling to the relative tilt angle) which is measurable by the sensor’ s integrated tilt measuring unit.

In order to describe the above mentioned complicated relationship [Equation (1)] analytically and accurately, in our former study [28], we have initially proposed the **EELA** model for I-type sensors. **EELA** has been tested to be a reliable and effective model to depict I-type sensors’ sensing properties.

However just as previously explained, it is a considerable limitations to suppose the vertical displacement does not vary or varies negligibly in the sliding deep displacement monitoring process, whether in theory or on practice. Therefore in this study, a II-type deep displacement sensor is proposed by making revisions to the structural design and the corresponding driver software of the I-type sensor.

**Figure 1.**
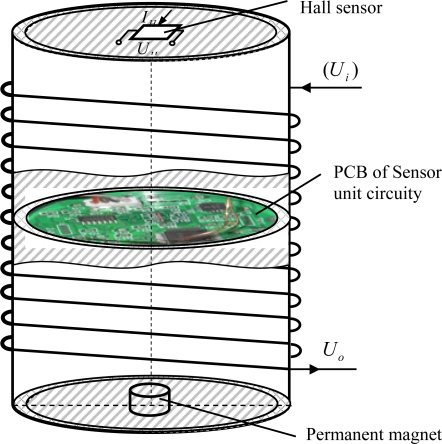
Structural diagram of a II-type deep displacement sensor unit.

Briefly speaking, the main structural revisions to each sensor unit of a II-type sensor includes a small cylindrical permanent magnet mounted at the center of the lower surface and a high sensitivity linear Hall sensor located at the center of upper surface (Figure 1). According to the Hall effect, the output voltage of Hall sensor in magnetic field satisfies the following change rule:

(2) $$U_{H}=R_{H}\;\frac{I_{H}\;B}{d}=R_{H}^{\prime }B$$

where *R_H_* and *d* are the Hall coefficient and semiconductor slice thickness of the Hall sensor respectively, *I_H_* is the current applied on the Hall sensor, *B* is the magnetic induction intensity exerted in the direction perpendicular to the upper surface of the sensor package.

**Figure 2.**
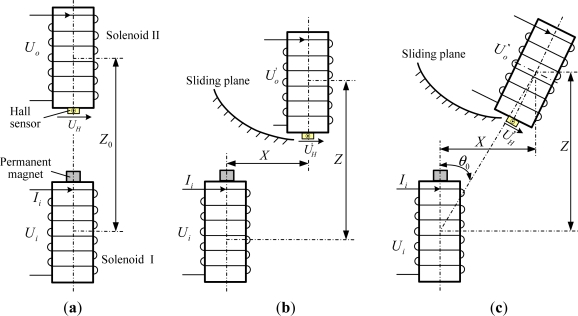
Geometrical arrangement between two arbitrary adjacent solenoids: (**a**) Initial arrangement; (**b**) Relative horizontal displacement and vertical displacement occurred; (**c**) Relative tilt, horizontal displacement and vertical displacement occurred.

As Figure 2 shows, when Solenoid I and II produce a relative displacement (whether horizontal displacement or vertical displacement or a combination of both), the relative position changes between the permanent magnet on the upper surface of Solenoid I and the Hall sensor on the lower surface of Solenoid II, so the magnetic field applied on the Hall sensor is changed accordingly, thus making the correlated Hall sensor output voltage change. That is, there exists a certain functional relationship between the magnetic induction intensity *B* and the relative position of Solenoid I and II. Considering the axial symmetry of cylindrical permanent magnet, the generated magnetic field also shows axial symmetry, which may be described as follows:

(3) $$B=f_{1}\;\left(X,\;Z,\;\theta_{0}\right)$$

where *X* and *Z* are the central distance and axial distance between Solenoid I and II and can be used to describe the relative horizontal displacement Ä*X* and relative vertical displacement Ä*Z*, respectively, *i.e*.,

(4) $$\begin{array}{l} \Delta X=X-X_{0}\\ \Delta Z=Z-Z_{0} \end{array}$$

where *X*_0_ and *Z*_0_ are the initial central distance and axial distance respectively between these two solenoids, generally setting *X*_0_ = 0 and *Z*_0_ = 40 mm.

There are some methods [32–35] to calculate the magnetic field **B** of a cylindrical permanent magnet, including the equivalent magnetic charge method, equivalent electric dipole method, equivalent current method and finite element method. Here we wouldn’t detail these due to the paper length limitations.

Combining Equations (2) and (3), the relationship between Solenoid II’s output Hall voltage *U_H_* and its position relative to Solenoid I can be described as:

(5) $$U_{H}=R_{H}^{\prime }\;f_{1}\;\left(X,\;Z,\;\theta_{0}\right)$$

Meanwhile, the functional relationship among Solenoid II’s output mutual inductance voltage *U_o_*, the relative geometrical position between Solenoid I and II in terms of relative horizontal displacement (Ä*X* = *X* − *X*_0_), vertical displacement (Ä*Z* = *Z* − *Z*_0_) and axial angle (*θ*_0_), and their geometrical parameters in terms of diameter *D*, length *A* and coil turn *W* can be expressed as:

(6) $$U_{o}=f_{2}\;\left(X,\;Z,\;\theta_{0},\;D,\;A,\;W\right)$$

Combing Equations (5) and (6), it shows that a II-type sensor requires no assumptions of no relative vertical displacement taking place between two adjacent solenoids during deep sliding monitoring process, so for any two adjacent sensor units, whether variations of relative horizontal displacement, vertical displacement, or axial angle, can cause the mutual inductance voltage *U_o_* and the Hall sensor output voltage *U_H_* to change at the same time. During the working process of II-type sensor, so long as *U_o_*, *U_H_* and *θ*_0_ between any two adjacent sensor units can be synchronously and automatically measured by the proposed sensor itself, and Equations (5) and (6) can be accurately expressed and evaluated by theoretical modeling or equation derivation, the said sensor can quantitatively determine the relative horizontal displacements and vertical displacements at different depths within the sliding mass. It is worth mentioning that the probable measured results include two special conditions: (i) the landslide is totally caused by horizontal displacement, then the sensor will measure Δ*Z* ≈ 0; (ii) the landslide is totally caused by vertical displacement, then the sensor will measure Δ*X* ≈ 0.

As mentioned above, the evaluation of Equation (5) is relatively simple with some available models and solving methods for reference. Compared to that, the evaluation of Equation (6) is much more complicated and remains a difficult issue for which there are few existing evaluation equations or models nowadays, so in this paper, one of the main theoretical tasks is to develop an efficient and accurate theoretical model to depict the complex functional relationship among mutual inductance voltage *U_o_*, the geometrical parameters of Solenoid I and II, and their relative central displacement *X*, axial distance *Z* and axial angle *θ*_0_, which reflect directly the relative sliding horizontal displacement, vertical displacement and tilt angle at the sliding mass’s corresponding depth.

It is worth stressing, just as our previous work has shown, that although both the change of mutual inductance voltage *U_o_* and that of mutual inductance *M* respond to the variations of relative displacement and axial angle between Solenoid I and II, it’s much simpler and more efficient to investigate deep displacement in terms of mutual inductance rather than mutual inductance voltage. Meanwhile, *M* is strictly proportional to *U_o_* and their relationship can be expressed as:

(7) $$U_{o}=\frac{1}{R}\;\frac{{\mathrm{dU}}_{i}}{\mathrm{dt}}\;M$$

where *R* is the equivalent resistance of Solenoid I, and *U_i_* is the 10 KHz sine voltage applied on Solenoid I.

Therefore, in this paper, we focus on II-type deep displacement sensor, and use the general Equation (8) to depict the functional relationship among mutual inductance *M*, the geometrical parameters of Solenoid I and II in terms of diameter *D*, length *A* and winding coil turns *W*, and their relative position in terms of central distance *X*, axial distance *Z* and axial angle *θ*_0_:

(8) $$M=f_{3}\;\left(X,\;Z,\;\theta_{0},\;D,\;A,\;W\right)$$

In order to evaluate the above complicated relationship [Equation (8)] qualitatively and effectively, we present in this paper a theoretical model called **NIELA** after extensive and intensive researches.

Compared to the existing **EELA** model, the proposed **NIELA** model uses a numerical integration approach rather than an equational derivation in infinite series form to evaluate the mutual inductance *M*, to meet the modeling requirement of varying both relative vertical displacement and horizontal displacement. Therefore **NIELA** is applicable not only for II-type sensors but also I-type sensors, whereas, **EELA**, it is generally only applicable for I-Type sensors, because the infinite series expressions for mutual inductance may be unconvergent and become invalid when varying the relative vertical displacement between Solenoid I and II.

## 3. NIELA for II-Type Deep Displacement Sensor

**EELA** was previously introduced in detail [28]. Here we only present a summary of this approach to explain why **EELA** is suitable for I-type sensors but not for II-Type sensors. Then we will introduce in detail **NIELA**, which is applicable both for I-type and II-type deep displacement sensors.

In brief, the **EELA** model is essentially an approximate calculation based on the double integration with the following basic steps:

*Step 1*: using two equivalent current loops to replace one solenoid, so the mutual inductance between any two adjacent solenoids can be equivalent as:
  (9) $$M=W^{2}\;\left(M_{13}+M_{14}+M_{23}+M_{24}\right)/4$$
  where *M*_13_, *M*_14_, *M*_23_ and *M*_24_ are the mutual inductances between two equivalent loops 1 and 3, 1 and 4, 2 and 3, 2 and 4, respectively.
*Step 2*: applying some related electromagnetic field theory and equations to deduce the equational expressions of mutual inductance for *M*_13_, *M*_14_, *M*_23_ and *M*_24_, respectively.

**Figure 3.**
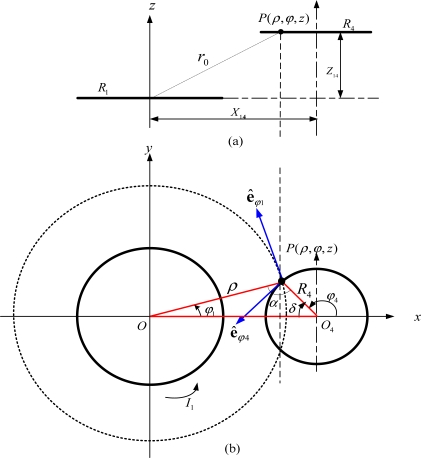
Geometric arrangement between equivalent parallel-axial Loop 2 and Loop 3. (**a**) Front view. (**b**) Top view.

For example, when Solenoid I and II are in parallel-axial arrangement, then *M*_13_, *M*_14_, *M*_23_ and *M*_24_ are the mutual inductances between two equivalent parallel-axial current loops 1 and 3, 1 and 4, 2 and 3, 2 and 4, respectively. When Loop *i* and Loop *j* are arranged as Figure 3 shown, we can deduce the following mutual inductance expression for *M_ij_*:

(10) $$M_{\mathrm{ij}}=\frac{\mu_{0}\pi D}{16}\;\sum_{n=1}^{\infty }\;{\left(-1\right)}^{n+1}\;\frac{n}{n+1}\;{\left[\frac{(2n-1)!!}{(2n)!!}\right]}^{2}\;\lambda_{\mathrm{ij}}^{2n+1}\;P_{2n}\;\left(\eta_{\mathrm{ij}}\right)$$

So long as the following convergence condition be satisfied:

(11) $$\lambda_{\mathrm{ij}}<1$$

where *i* = {1, 2}, *j* = {3, 4}, *R_i_* = *R_j_* = *D*/2, $r_{\mathrm{ij}}=\sqrt{X_{\mathrm{ij}}^{2}+Z_{\mathrm{ij}}^{2}}$, *λ* = *D*/*r_ij_*, *η_ij_* = *Z_ij_*/*r_ij_*, *P*_2*n*_(*η_ij_*) is the Legendre polynomials of degree 2*n* with argument *η_ij_*, and *μ*_0_ = 4*π*×10^−7^ *H / m* is the free space permeability.

Obviously, the expression for *M_ij_* is quite complicated and expressed by an infinite series, which means, when *λ_ij_* << 1, *M_ij_* is dominated by the first several terms because the series converges quickly, but the more *λ_ij_* is close to 1, the more slowly the series converges, and the more terms need be calculated to get an approximation to *M_ij_* with sufficient accuracy, so calculation of *M*_14_ is very time-consuming when *λ_ij_* is very near 1. Much worse, when *λ_ij_* happens to be 1 or larger than 1, then Equation (10) cannot converge at all.

From Equation (11), it is seen that, under a given set of conditions, *λ_ij_* will be smaller if *r_ij_* is larger. When Solenoid I and II are in parallel state, *X_ij_* is equal to *X*, which is determined by the relative horizontal displacement between Solenoid I and II that occurred along with the slope movement. That’s to say, when the horizontal movement of slope is very slow or the sensor is buried in the slope mass not long ago, *X_ij_* may be so small (for instance, *X_ij_* = 5–10 mm) that *r_ij_* is mainly determined by *Z_ij_*. Note that in these four equivalent loops, Loop 2 and 3 are the closest to each other, so *Z*_23_ is the smallest one of *Z_ij_*(*Z*_13_, *Z*_14_, *Z*_23_, *Z*_24_), that is, if *Z*_23_ satisfies the convergence condition, then the other three *Z_ij_* are sure to converge. Now we will examine *Z*_23_ in detail.

For the experimental sensor prototype, the solenoids’ diameter and length are set to be *D* = 70 mm and *A* = 75 mm. According to the equivalent loop approach, approximately $Z_{23}=Z-A/\sqrt{3}$. Then the convergence condition for *Z* can be expressed by:

(12) $$Z>\sqrt{D^{2}-X_{23}^{2}}+A/\sqrt{3}$$

For example, if *X* = 5 mm, *Z* must be larger than 113.1 mm; if *X* = 10 mm, *Z* must be larger than 112.6 mm, and so on.

So for an I-type sensor and the correlated experiments conducted before [28], the initial value of *Z* was set as 115 mm and supposed to not vary with the sliding movement. Under such an arrangement, *λ*_23_ and all other *λ_ij_* could be guaranteed to converge, so Equation (10) could be quickly convergent and thus effective in calculation. From this it follows that **EELA** is applicable to I-type sensors.

Furthermore, after a series of comparisons and examinations conducted [28] between the predicted mutual inductance based on **EELA** and the experimentally measured mutual inductance voltage based on an I-type sensor prototype, the **EELA** model was tested to be reliable and effective in depicting an I-type sensor’s sensing properties (*i.e*., determining the relative horizontal displacement and tilt angle quantitatively) with acceptable estimation accuracy on the premise of convergence.

However, considering there exist limitations in assuming the vertical displacement does not vary in the sliding process whether in theory or on practice, the II-type deep displacement sensor is proposed to free us from this assumption. That is, for a II-type sensor, any two adjacent sensor units are free to relatively tilt (*θ*_0_), move horizontally (*X*), and move vertically (*Z*) along with sliding of the surrounding rock and soil mass.

Under such instances, when *Z* is reduced from the initial 115 mm to 110 mm or less, Equation (10) will no longer converge and becomes invalid to evaluate *M_ij_*. When Solenoid I and II are arranged in cross-axial state, the equational expressions deduced for *M_ij_* are also complicated and form an infinite series, which we have derived in detail before [28]. All these facts show that **EELA** is basically invalid to depict II-type sensors due to the non-convergence problem during variation of relative vertical displacement between any two adjacent sensor units.

In this paper, a new theoretical modeling named *numerical integration-based equivalent loop approach* (**NIELA**) is proposed to depict the mutual inductance properties of II-type sensor. This model can qualitatively depict the complicated relationships among mutual inductance *M*, geometrical parameters of Solenoid I and II in terms of diameter *D*, length *A* and coil turns *W*, and their relative position in terms of relative horizontal displacement (Ä*X* = *X* − *X*_0_), vertical displacement (Ä*Z* = *Z* − *Z*_0_) and axial angle (*θ*_0_) for any two adjacent sensor units just as Equation (9) denoted.

Compared to **EELA**, **NIELA** applies the same hypotheses for the modeled air-core solenoids [28] and the same equation [*i.e*., Equation (9)] to evaluate the mutual inductance between any two adjacent solenoids, but uses numerical integration rather than infinite series to express and evaluate *M*_13_, *M*_14_, *M*_23_ and *M*_24_. For convenience of interpretation, we will demonstrate how *M*_14_ is evaluated in the **NIELA** model when Loop 1 and 4 are arranged in a parallel-axial state as shown in Figure 3.

Firstly, the Cartesian coordinate *O-xyz* and polar coordinate *O-ρφz* are established simultaneously, in which we let Loop 1 and Loop 4 lie in the *xy* plane, having radii *R*_1_ and *R*_4_, respectively, and apart from each other by an axial distance *Z*_14_ and a central distance *X*_14_. Supposing Loop 1 carries current *I*_1_, then under the polar coordinate system, an arbitrary source point *Q* in Loop 1 can be denoted as *Q*(*ρ*_1_,*φ*_1_,*Z*_1_) = *Q*(*R*_1_,*φ*_1_, 0), and according to the Biot-Savart law, the vector potential at an arbitrary field point *P*(*ρ, φ, z*) due to current *I*_1_ in Loop 1 is:

(13) $$A_{1}(\rho ,\;\varphi ,\;z)=\frac{\mu_{0}}{4\pi }\;\int_{c}\;\frac{I_{1}d\ell }{R}$$

In Equation (13), the integration is along the direction of current flow, the current element *I*_1_*d*ℓ is tangent to Loop 1 at source point *Q*, **R** is the distance vector from the source *d*ℓ to the field point *P*, *R* = |**R**| and **R̂** = **R**/*R*, *μ*_0_ = 4*π*×10^−7^ *H*/*m* is the free space permeability. After simplification:

(14) $$A_{1}(\rho ,\;\varphi ,\;z)=A_{\rho 1}\;{\hat{e}}_{\rho }+A_{\varphi 1}\;{\hat{e}}_{\varphi 1}$$

(15) $$A_{\rho 1}=\frac{\mu_{0}\;I_{1}\;R_{1}}{4\pi }\;\int_{-\pi }^{\pi }\;\frac{\text{sin}\;\phi d\phi }{\sqrt{\rho^{2}+R_{1}^{2}-2\rho R_{1}\;\text{cos}\;\phi +z^{2}}}d\phi$$

(16) $$A_{\varphi 1}=\frac{\mu_{0}\;I_{1}\;R_{1}}{4\pi }\;\int_{-\pi }^{\pi }\;\frac{\text{cos}\;\phi d\phi }{\sqrt{\rho^{2}+R_{1}^{2}-2\rho R_{1}\;\text{cos}\;\phi +z^{2}}}d\phi$$

where *ϕ* = *φ*−*φ*_1_. Using parity of trigonometric functions, we can prove the integral of *ρ* component of Equation (14), *A_ρ_*_1_ = 0, so the vector potential is azimuthal, *i.e*., **A**_1_(*ρ*,*φ*,*z*) = *A*_*φ*1_(*ρ*,*z*)**ê**_**φ**1_. Let *ϕ* = *π* − 2*β*, then:

(17) $$\begin{array}{cc} A_{\varphi 1}\;(\rho ,\;z) & =\frac{\mu_{0}\;I_{1}\;R_{1}}{\pi }\;\int_{0}^{\frac{\pi }{2}}\;\frac{2\;{\text{sin}}^{2}\;\beta -1}{\sqrt{{\left(\rho +R_{1}\right)}^{2}+z^{2}-4\rho R_{1}\;{\text{sin}}^{2}\;\beta }}d\beta \\ & =\frac{\mu_{0}\;I_{1}\;R_{1}}{\pi \sqrt{{\left(\rho +R_{1}\right)}^{2}+z^{2}}}\;\int_{0}^{\frac{\pi }{2}}\;\frac{2\;{\text{sin}}^{2}\;\beta -1}{\sqrt{1-k^{2}\;{\text{sin}}^{2}\;\beta }}d\;\beta \\ & =\mu_{0}\;I_{1}\;\sqrt{R_{1}/\rho }f(k)/(2k) \end{array}$$

and:

(18) f (k)=(2/k−k) K (k)−2E (k)/k

where $k=\sqrt{4\rho R_{1}/\left[{\left(\rho +R_{1}\right)}^{2}+z^{2}\right]}$, *K* and *E* are the complete elliptic integrals of the first and second kinds respectively with modulus *k*:

(19) $$K\;(k)=\int_{0}^{\pi /2}\;\frac{d\;\beta }{\sqrt{1-k^{2}\;{\text{sin}}^{2}\;\beta }}$$

(20) $$E\;(k)=\int_{0}^{\pi /2}\;\sqrt{1-k^{2}\;{\text{sin}}^{2}\;\beta }d\beta$$

If we limit *P* to be one point in Loop 4 as Figure 3 shown, then:

(21) $$P\;(\rho ,\;\varphi ,\;z)=P\left(\rho_{4},\;\varphi_{4},\;z_{4}\right)$$

(22) $$\;\rho_{4}=\sqrt{X_{14}^{2}+R_{4}^{2}+2X_{14}R_{4}\;\text{cos}\;\varphi_{4}}$$

(23) $$\text{cos}\;\alpha =\frac{\rho_{4}^{2}+R_{4}^{2}-X_{14}^{2}}{2R_{4}\rho_{4}}=\frac{R_{4}+X_{14}\;\text{cos}\;\varphi_{4}}{R_{4}\rho_{4}}$$

(24) $$z_{4}=Z_{14}$$

According to the electromagnetic induction theory, the mutual inductance between Loop 1 and Loop 4, *M*_41_ ≡ Φ_41_/*I*_1_, where Φ_41_ is the magnetic flux through Loop 4 due to current *I*_1_ in Loop 1. And Φ_41_ can be evaluated by:

(25) $$\Phi_{41}=\oint_{C4}\;A_{1}(P)\cdot d\ell_{4}$$

In our case:

(26) $$\begin{array}{ll} M_{14} & =\frac{1}{I_{1}}\;\oint_{C4}\;A_{\varphi 1}\;{\hat{e}}_{\varphi 1}\cdot {\hat{e}}_{\varphi 4}\;{\mathrm{dC}}_{4}\\ & =\frac{1}{I_{1}}\;\int_{0}^{2\pi }\;A_{\varphi 1}\;\left(\rho_{4},\;Z_{14}\right)\;R_{4}\;\text{cos}\;\alpha d\varphi_{4} \end{array}$$

To evaluate *M*_14_ explicitly, we first calculate *M*_0_, the mutual inductance between two coaxial current loops whose radii are *R*_1_ and *ρ*_4_ respectively, and *Z*_14_ apart in *z* axis:

(27) $$M_{0}=\frac{1}{I_{1}}\;\int_{0}^{2\pi }\;A_{\varphi 1}\;(\rho ,\;z){\hat{e}}_{\varphi 1}\;\cdot {\hat{e}}_{\varphi 1}\;\rho d\varphi_{1}=\frac{2\pi \rho_{4}}{I_{1}}\;A_{\varphi 1}\;\left(\rho_{4},\;Z_{14}\right)$$

Then *M*_14_ can be associated with *M*_0_ by:

(28) $$\begin{array}{c} M_{14}=\int_{0}^{2\pi }\;\left[2\pi \rho_{4}\;A_{\varphi 1}\;\left(\rho_{4},\;Z_{14}\right)/I_{1}\right]\;\left[R_{4}\;\text{cos}\;\alpha /\left(2\pi \rho_{4}\right)\right]\;\;d\varphi_{4}\\ =\int_{0}^{2\pi }\;M_{0}\left[R_{4}\;\text{cos}\;\alpha /\left(2\pi \rho_{4}\right)\right]d\varphi_{4} \end{array}$$

Let *φ*=*φ*_4_, then:

(29) $$M_{14}=M_{41}=\frac{1}{\pi }\;\int_{0}^{\pi }\;\frac{M_{0}\;R_{4}\;\text{cos}\;\alpha }{\pi }d\varphi$$

(30) $$\text{cos}\;\alpha =\left(R_{4}+X_{14}\;\text{cos}\;\varphi \right)/\left(R_{4}\;\rho_{4}\right)$$

Combining Equation (29) with Equations (17–20), (22), (27), (28), and (30), *M*_14_ can be easily evaluated by numerical integration over the range 0 ≤ *φ* ≤ *π*. From this, we can see the convergence limitations set upon **EELA** has been completely overcome by **NIELA**, so **NIELA** is applicable for the proposed II-type deep displacement sensor.

## 4. Experimental Testing and Model Verification

### 4.1. Experimental Setup and Procedure

**Figure 4.**
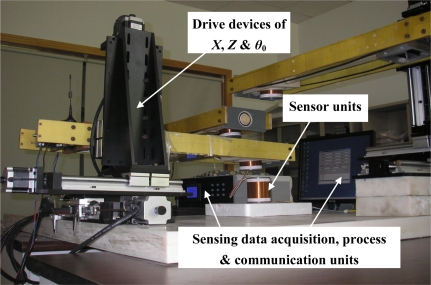
Photograph of the sensor prototype based experimental setup.

To verify the above analysis and to test the performance of the proposed **NIELA** method on evaluating of the mutual inductance *versus* the horizontal displacement and vertical displacement between two adjacent sensor units for a II-type sensor, we conducted a series of experiments and comparisons using a sensor prototype and some related devices, which include the sensor’s axial, horizontal displacement and vertical displacement drive devices, axial angle measurement unit, sensing data acquisition, processing, communication and display unit, *etc*. The sensor prototype mainly includes two adjacent integrated deep displacement sensor units (Solenoid I and II) and a deep displacement measuring central processing unit. Under the control of the central processing unit, the sine input voltage *U_i_* can be automatically generated on Solenoid I, the corresponded mutual inductance voltage *U_o_* across Solenoid II and the axial distance *Z*, central distance *X* and axial tilt angle *θ*_0_ between them can be automatically measured and recorded in real time and further transmitted to a remote or local comprehensive processing center for detailed process through RS-485 or wireless communication. *X*, *Z* and *θ*_0_ can consecutively adjusted by the sensor’s axial, horizontal displacement and vertical displacement drive devices. The detailed sensor fabrication process and supported devices arrangement have been introduced before [28] and a photograph of the experimental setup is shown in Figure 4.

Model verification process mainly includes two parts: (i) test the modeling effectiveness of **EELA** and **NIELA** for an I-type sensor (*X* Variable, *Z* invariant); (ii) test the modeling effectiveness of **EELA** and **NIELA** for a II-type sensor (both *X* and *Z* Variable). This is conducted mainly by comparison among the measured mutual inductance voltage, **NIELA**-based mutual inductance, **EELA**-based mutual inductance under the same given conditions. It is noted that the change of mutual inductance should be completely proportional to mutual inductance voltage in theory [28].

### 4.2. Experiments and Model Validation One (*Z* not varied)

To test the modeling effectiveness of **NIELA** and **EELA** for an I-type sensor (*X* variable, *Z* invariant), we first conducted experiments using the following assumptions: under the impact of deep slope movement, both the relative horizontal displacement *X* and tilt angle *θ*_0_ are changed between Solenoid I and II, but their relative vertical displacement (Ä*Z* = *Z* − *Z*_0_) does not vary or varies negligibly.

**Figure 5.**
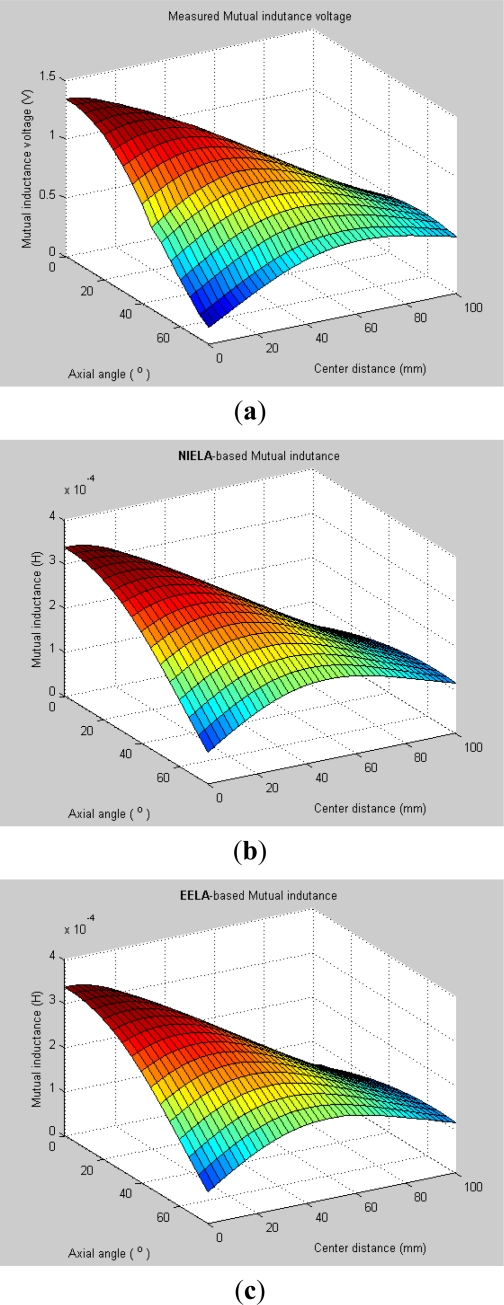
3-D graphs of (**a**) measured mutual inductance voltage; (**b**) **NIELA**-based mutual inductance; (**c**) **EELA**-based mutual inductance *versus* center distance and axial angle between Solenoid I and II (*Z* = 115 mm).

Obviously, an I-type sensor is workable in such instances, so in the experiment, we fixed the axial distance *Z* to be 115 mm (*i.e*., *Z* = *Z*_0_) but gradually varied *X* (0–100 mm, range interval: Δ*X* = 2.5 mm) and *θ*_0_ (0–75°, range interval: Δ*θ* = 5°), and recorded the corresponding output of the mutual inductance voltage between these two solenoids, and finally plotted these measured data into 3-D graphs, as shown in Figure 5(a). Meanwhile, we plotted the corresponding 3-D theoretical prediction graphs based on **NIELA** and **EELA** respectively, which are shown in Figure 5(b,c). The geometrical parameters for the modeled and sensor prototype-based solenoids are listed in Table 1. A comparison of Figure 5(b,c) to Figure 5(a) shows that very good agreements are achieved between the experimental data and modeling output wherever Solenoid I and II are in coaxial, parallel-axial or cross-axial states, which indicates both the **NIELA** and **EELA** models are quite reliable and effective to describe the property of an I-type deep displacement sensor.

**Figure 6.**
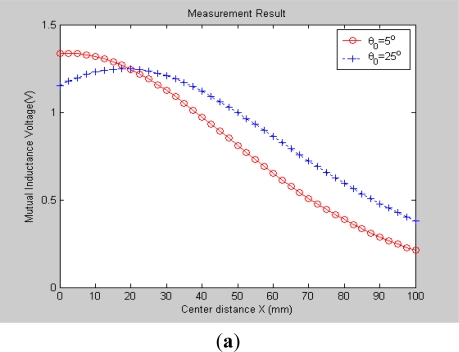

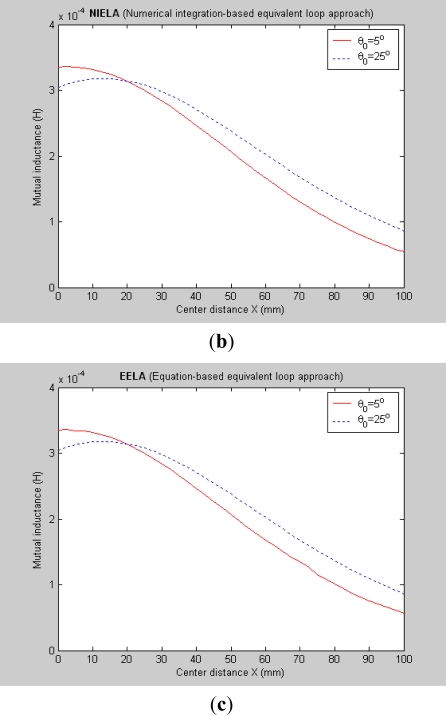
2-D graphs of (**a**) measured mutual inductance voltage; (**b**) **NIELA**-based mutual inductance; (**c**) **EELA**-based mutual inductance *versus* center distance between Solenoid I and II (*Z* = 115 mm).

To allow further examinations, some 2-D curves were extracted from its 3-D graphs in Figure 5 by specifying some axial angle *θ*_0_ (for instance, 5° and 25°) as Figure 6 shows. A comparison between Figure 6(a) and Figure 6(b,c) shows that the experimental data still show high shape similarity to modeling results based on both **NIELA** and **EELA**, thereby further verifying these two models’ reliability and high approximation in formulation of an I-type sensor’s sensing properties under the hypothesis that no relative vertical displacement occurred between any two adjacent sensor units inside the sliding mass.

### 4.3. Experiments and Model Validation Two (Z varied)

As we know, to suppose the vertical displacement does not change when landslides and related geo-engineering happens does not fully meet the practical situation of sliding movement, so in this part, we will study the influence of both the deep vertical displacement and deep horizontal displacement (*X* & *Z* variable) on the sliding mass and fully examine the modeling effectiveness of **NIELA** for a II-type sensor under such circumstances. A series of comparative experiments were conducted among the measured mutual inductance voltage, the predicted mutual inductance based on **NIELA** and **EELA** respectively, *versus* the simultaneous variation of axial distance *Z* and central distance *X* under some fixed axial angers *θ*_0_ (*θ*_0_ can be automatically measured by a II-type sensor) between Solenoid I and II.

**Figure 7.**
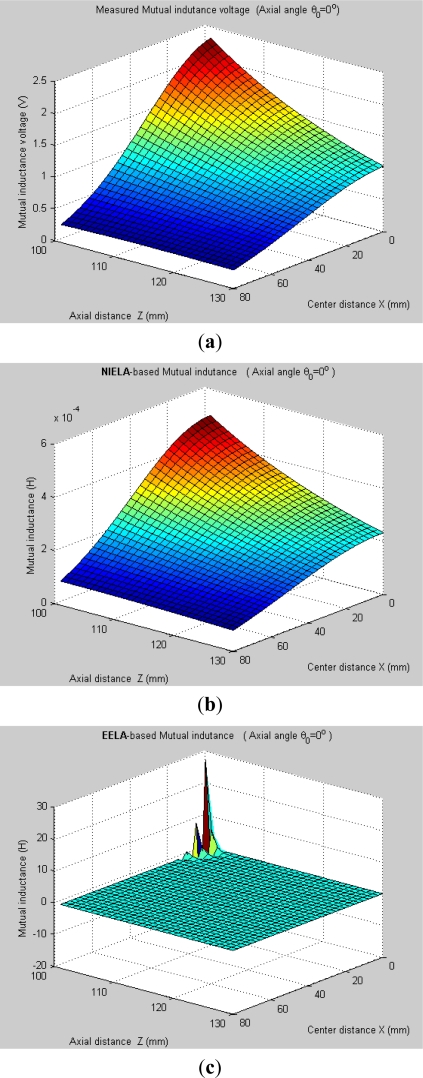
3-D graphs of (**a**) measured mutual inductance voltage; (**b**) **NIELA**-based mutual inductance; (**c**) **EELA**-based mutual inductance *versus* axial distance and center distance (*θ*_0_ = 0°, *Z* = 101–130 mm).

**Figure 8.**
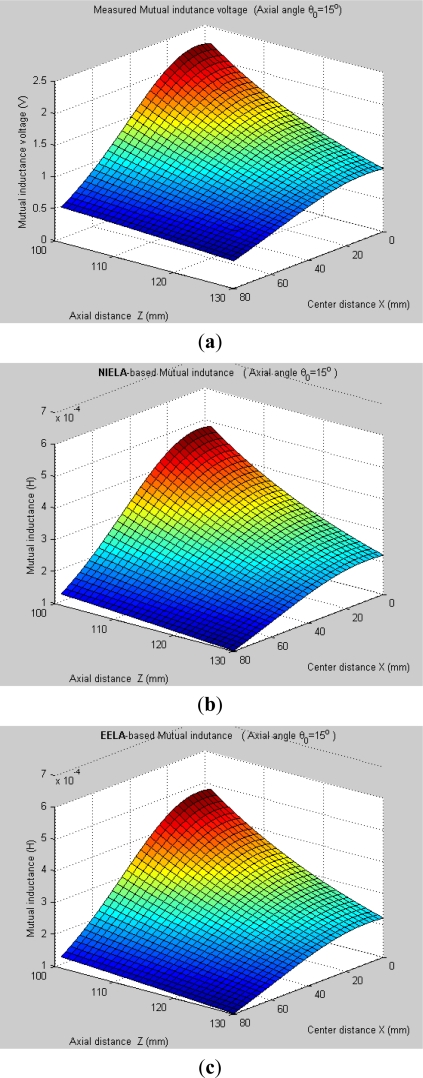
3-D graphs of (**a**) measured mutual inductance voltage; (**b**) **NIELA**-based mutual inductance; (**c**) **EELA**-based mutual inductance *versus* axial distance and center distance (*θ*_0_ = 15°, *Z* = 101–130 mm).

It can be seen, Figures 7 and 8 plot the 3-D graphs of (a) measured mutual inductance voltage, (b) **NIELA**-based mutual inductance and (c) **EELA**-based mutual inductance *versus* center distance *X* and axial distance *Z* between Solenoid I and II under two different conditions, respectively:

*Condition 1*: *θ*_0_ = 0°, *Z* = 101–130 mm
*Condition 2*: *θ*_0_ ≠ 0°, *Z* = 101–130 mm

**Figure 9.**
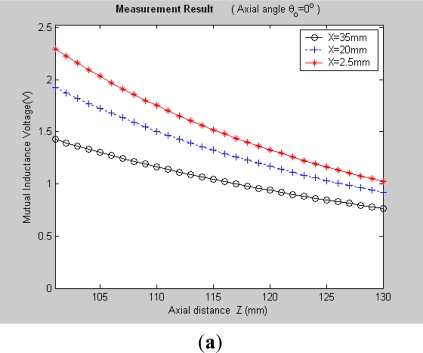

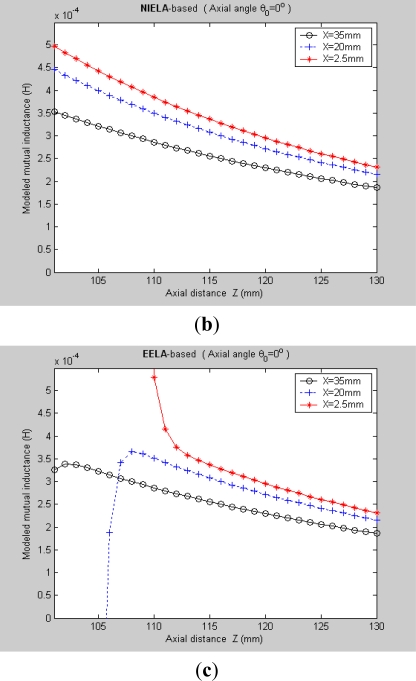
2-D graphs of (**a**) measured mutual inductance voltage; (**b**) **NIELA**-based mutual inductance; (**c**) **EELA**-based mutual inductance *versus* axial distance *Z* (*θ*_0_ = 0°, *Z* = 101–130 mm).

**Figure 10.**
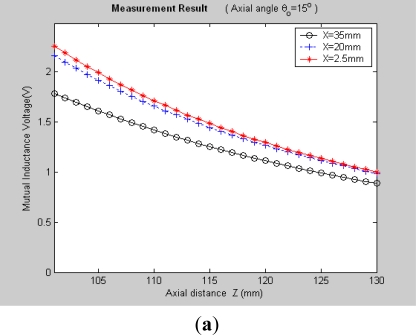

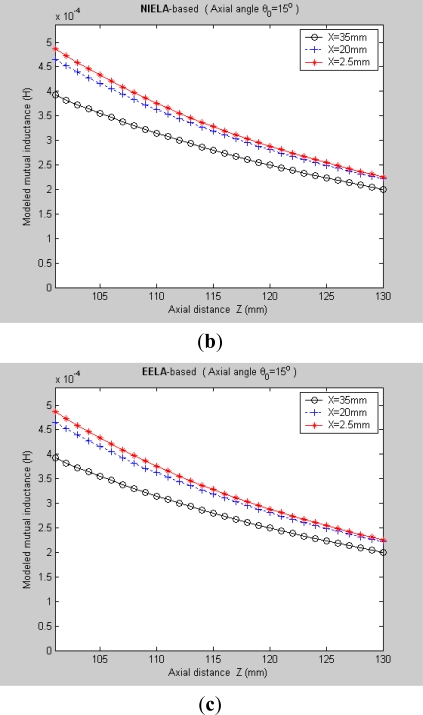
2-D graphs of (**a**) measured mutual inductance voltage; (**b**) **NIELA**-based mutual inductance; (**c**) **EELA**-based mutual inductance *versus* axial distance *Z* (*θ*_0_ = 15°, *Z* = 101–130 mm).

A series of comparative studies show that:

- Under Condition 1, where the convergence conditions for **EELA** cannot be satisfied, a great discrepancy has occurred between the measured mutual inductance voltage and the **EELA**-predicted mutual inductance, so **EELA** is shown to be invalid due to its divergence, whereas, under the same conditions, the **NIELA**-based mutual inductance shows high consistency to the measured mutual inductance voltage, so the **NIELA** model is tested to be valid and effective under such conditions.
- Under Condition 2, where **EELA** satisfies the convergence conditions, both the **NIELA**-based and **EELA**-based mutual inductances show good tracking of the measured results of mutual inductance voltage, so **NIELA** is still verified to be feasible and effective in modeling a II-type sensor under condition 2. **EELA** also seems effective under this condition.
- To allow further studies, as shown in Figures 9 and 10, from the 3-D graphs in Figures 7 and 8, we have extracted some 2-D curves by fixing the value of *X*, which offers a close-up view of the effect of axial distance *Z* on the measured mutual inductance voltage and predicted mutual inductance based on **NIELA** and **EELA,** respectively, under some specific central distance *X*. Figures 9 and 10 show the simulation results in parallel-axial and cross-axial state, respectively.

It is noted that, only with the premise of convergence could **EELA** be correctly apply to theoretical modeling for the deep displacement sensor. That is, when *Z* is smaller than a certain fixed value (*i.e*., convergence limit), the convergence condition for **EELA** could no longer be satisfied, so the predicted mutual inductance is meaningless and invalid, which has been clearly demonstrated by Figure 9(c). Meanwhile, the **NIELA**-based 2-D theoretical curves [Figure 9(b)] are seen to be quite in agreement with the experimental one [Figure 9(a)] wherever *X* = 2.5 mm, 20 mm or 35 mm.

**Figure 11.**
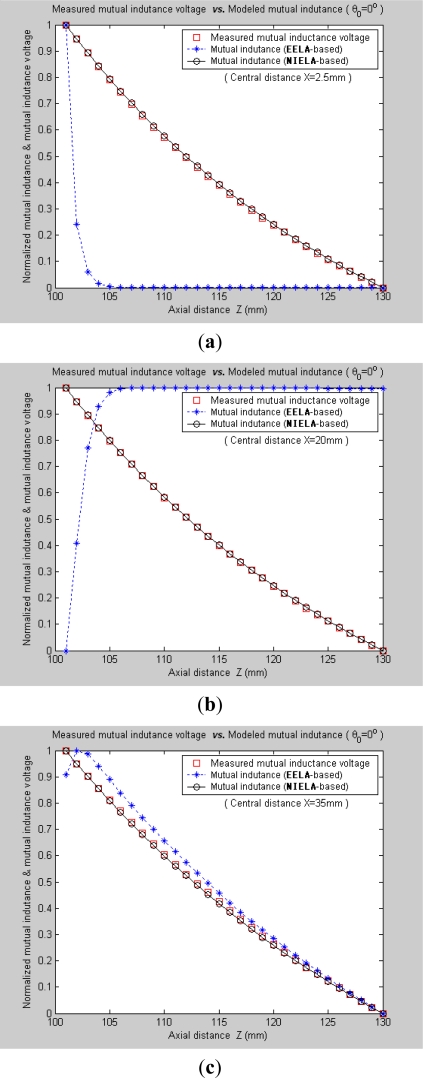
Normalized curves for measured mutual inductance voltage, **NIELA**-based mutual inductance and **EELA**-based mutual inductance when *θ*_0_ = 0°. (**a**) *X* = 2.5 mm; (**b**) *X* = 20 mm; (**c**) *X* = 35 mm.

**Figure 12.**
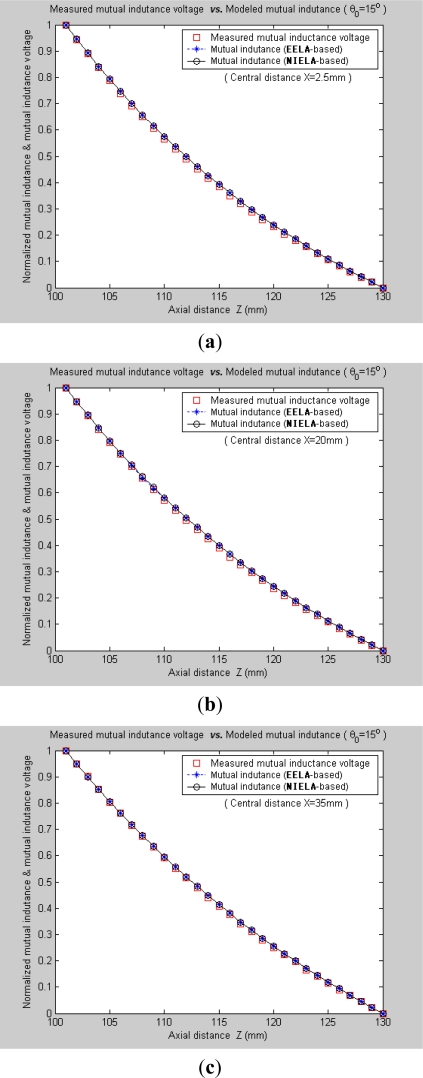
Normalized curves for measured mutual inductance voltage, **NIELA**-based mutual inductance and **EELA**-based mutual inductance when *θ*_0_ = 15°. (**a**) *X* = 2.5 mm; (**b**) *X* = 20 mm; (**c**) *X* = 35 mm.

Figure 11(a–c) further plot the normalized curves of the measured mutual inductance voltage, **NIELA**-based mutual inductance, and **EELA**-based mutual inductance respectively according to Figure 9. It can be seen that, under such stringent point to point spatial comparison, the **NIELA**-based mutual inductance still shows good tracking of the measured mutual inductance voltage, which further increases our confidence in using the proposed **NIELA** model to predict both horizontal displacement and vertical displacement variations for the II-type sensor. However for **EELA**, so long as the convergence conditions are not met, its normalized curves of mutual inductance show little shape similarity to the measured one, so the **EELA** model becomes invalid and unqualified for II-type sensors under such instances. As can be seen from Figure 10, under cross-axial state, both **NIELA**-based and **EELA**-based mutual inductance match the actual mutual inductance voltage very well. In the same way, we have further plot the normalized curves from Figure 10 and labeled them as Figure 12, which shows that even under such stringent point to point spatial comparison, these three normalized curves, that is, **NIELA**-based predictions, **EELA**-based predictions and sensor prototype-based measurement results, are almost completely overlapped wherever *X* = 2.5 mm, 20 mm or 35 mm.

This further validates that **NIELA** models could quite accurately estimate the variations of both horizontal displacement and vertical displacement during sliding movement for the proposed II-type deep displacement sensor, whether any two adjacent sensor units relatively slide horizontally, vertically or tilt.

## 5. Conclusions

Landslides are one of the most costly catastrophic events in terms of human lives and property losses. Deep displacement monitoring is one basic means of dynamic study and early warning monitoring of landslides. It is also an important part of engineering geological investigation. Presently, there are few single sensors or instruments that can simultaneously and efficiently monitor the deep horizontal displacements and vertical displacements from surface to different depths within the monitored soil and rock mass on purpose of sliding geohazard monitoring or treatment engineering assessment.

In our previous work, we have proposed an electromagnetic induction-based deep displacement sensor (I-type deep displacement sensor) and a corresponding theoretical model called **EELA** to predict the deep horizontal displacement at different depths within the landslide mass.

In this study, in order to meet the engineering requirement of monitoring both the horizontal displacement and vertical displacement at different depths within the sliding mass, the II-type deep displacement sensor is proposed by modifying the I-type sensor. Compared to the I-type sensor, whether the variations of relative horizontal displacement, vertical displacement, or axial angle between any two adjacent sense units, can cause the mutual inductance voltage *U_o_* (which is proportional to mutual inductance *M*) and the Hall sensor output voltage *U_H_* to vary simultaneously, so a II-type sensor need not make assumptions that no relative vertical displacement occurred inside the slope mass. In all, the proposed II-type sensor combines deep horizontal displacement and vertical displacement monitoring capability.

To depict a II-type sensor’s mutual inductance properties analytically and quantitatively, a theoretical model called *numerical integration-based equivalent loop approach* (**NIELA**) is presented. Combining numerical integration technique with equivalent loop approach, this model can quite accurately evaluate the complicated relationship among the mutual inductance, the geometrical parameters of any two adjacent sensor units, and their relative position (*i.e.*, horizontal displacement, vertical displacement and tilt angle) just as Equation (8) denoted, through which to predict both deep horizontal displacement and vertical displacement variations for a II-type sensor.

To test the **NIELA** model’s theoretical reliability and estimation accuracy for the proposed II-type sensor, a series of comparisons and examinations have been conducted between the measured mutual inductance voltage, **NIELA**-based mutual inductance and **EELA**-based mutual inductance under several application circumstances, from which some main conclusions can be drawn as follows:

1. “Experiments and Model Validation One” (where *Z* is assumed to not vary during slide process so the convergent condition for **EELA** can be guaranteed) shows that very good agreements have been achieved among the experimentally measured data, **NIELA**-based predictions and **EELA**-based predictions, which indicates both **NIELA** and **EELA** can effectively and quantitatively express the sensing properties of an I-type deep displacement sensor.
2. Through “Experiments and Model Validation Two” (where both *X* and *Z* varied), we can see that: (i) a great discrepancy has occurred between the measured mutual inductance voltage and **EELA**-based predicted mutual inductance due to a lack of convergence for **EELA** when varying the relative vertical displacement between any two adjacent senor units, so the **EELA** model is tested to be basically invalid for the II-type sensor due to the convergence limitations. Secondly, the **NIELA**-based mutual inductance is found to be in good agreement with the measured mutual inductance voltage, which indicates that **NIELA** is a relatively accurate and efficient model to predict both the deep horizontal displacement and vertical displacement for the proposed II-type sensor.
3. In sum, the **NIELA**-based predicted mutual inductance always shows good tracking of the measured mutual inductance voltage under all conditions in any experiments conducted, even including the most stringent point to point spatial comparisons between them. It can be said that all experiments conducted here have verified the **NIELA** model’s high theoretical reliability and prediction accuracy in depicting of the mutual inductance characters of II-type deep displacement sensor, so both the deep horizontal displacement and vertical displacement at different depths within the slope mass could be quantitatively predicted.

These conclusions, in turns, support these two opinions:

1. **EELA** is well qualified to describe the sensing characters of an I-type deep displacement sensor, which is mainly applied to monitor such landslides and related geo-engineering whose main form of movement is horizontal displacement while the vertical movement is relatively small or unimportant.
2. **NIELA** is a quite reliable and high approximation model to describe the sensing properties both for I-type and II-type deep displacement sensors, so it is generally applicable for monitoring of different kinds of landslides and some related geo-engineering problems, especially for such monitoring circumstances that both the underground vertical displacement and horizontal displacement change dynamically during the sliding process thus a simultaneous monitor toward both displacements may really required.

## Figures and Tables

**Table 1.** Geometrical parameters of Solenoid I and II.

**Parameter**	**Unit**	**Value**	**Comment**
Diameter (*D*)	mm	70	
Length (*A*)	mm	75	
Axial distance (*Z*)	mm	115	
Coil turns (*W*)	mm	400	divided by 3 layers
